# Prevalence and Associated Factors of Overweight and Obesity among High School Adolescents in Bahir Dar City, Northwest, Ethiopia: A Cross-Sectional Study

**DOI:** 10.1155/2021/8846723

**Published:** 2021-03-09

**Authors:** Mulugebeya Worku, Zemichael Gizaw, Aysheshim Kassahun Belew, Alemakef Wagnew, Melkamu Tamir Hunegnaw

**Affiliations:** ^1^Department of Human Nutrition, Institute of Public Health, University of Gondar, Gondar, Ethiopia; ^2^Department of Environmental and Occupational Health, Institute of Public Health, University of Gondar, Gondar, Ethiopia; ^3^Department of Biostatistics and Epidemiology, Institute of Public Health, University of Gondar, Gondar, Ethiopia

## Abstract

**Background:**

Overweight and obesity can be defined as excessive and abnormal fat deposition in our bodies. A body mass index for age of Z scores +2 to +3 was classified as overweight, whereas BMI for age >+3 Z-score was considered as obesity. Overweight and obesity in adolescents are a major health problem in low-income countries like Ethiopia. However, there is not well-established data on adolescents' overweight and obesity. Therefore, this study assessed the prevalence and associated factors of overweight and obesity among high school adolescents in Bahir Dar city, northwest of Ethiopia.

**Methods:**

An institution-based cross-sectional study was conducted from October 30 to November 30/2019 among 551 high school adolescents. Data were collected using a self-administrative questionnaire. Weight and height were measured by trained health professionals. World Health Organization AnthroPlus software was used to analyze anthropometric data into body mass index for age with z-score to ascertained overweight and obesity. Data were entered using Epi Info version 3.5.3 and transferred to SPSS version 22 for further analysis. Frequency and percentage were presented using tables and figures. A bivariable and multivariable logistic regression analysis was performed to determine the association between the dependent and independent variables. Adjusted odds ratio with 95% CI and *p* < 0.05 were used to dictate statistical significance for overweight and obesity.

**Result:**

In this study, 522 high school adolescents aged 10–19 years were selected using a simple random sampling technique with a response rate of 94.74%. The mean age of the respondents was 17 years with SD ± 1.41. The overall prevalence of overweight and obesity was 12.5% (95% CI: 9.6, 15.2). Males (13.3%) were more than females (11.5%), being overweight and obese. Having self-employed mothers (AOR: 4.57; 95% CI: 1.06, 19.78), having government-employed mothers (AOR: 6.49; 95% CI: 1.96, 21.54), and having school feeding habit (AOR: 0.44; 95% CI: 0.26, 0.76) were factors associated with overweight/obesity among high school adolescents.

**Conclusion:**

The prevalence of overweight/obesity in the current study was high. Adolescents having self-employed mothers, adolescents having government-employed mothers, and students having school feeding habits were significant factors of overweight and obesity. Therefore, more emphasis will be given to adolescents having self- and government-employed mothers and adolescents having school feeding habit.

## 1. Background

Overweight and obesity are defined as abnormal or excessive fat accumulation, resulting in weakening the health of an individual [1–3]. The epidemic of overweight and obesity reflects the changes in society and behavioral patterns of communities over recent decades. Even if genes are important in determining a person's susceptibility to weight gain, energy balance is also a factor for overweight and obesity. Societal changes and worldwide nutrition transition are driving the overweight/obesity epidemic [4, 5]. Adolescence is a vulnerable period for the development of obesity and the weight of adolescent tracks strongly into adulthood [6]. Adolescent overweight and obesity are increasing globally, raising the threat of long-term illness in later adulthood [7,8].

Overweight and obesity are a result of an imbalance between energy intake and expenditure with an increase in energy balance being closely associated with the lifestyle adopted and the dietary intake preferences [9]. Adolescents affected by overweight and obesity are predisposed to various morbidities including neuropathy, retinopathy, nephropathy, hypertension, and dyslipidemia, which in turn also increases the rate of mortality in the adulthood period [10–12]. In addition, adolescents' overweight and obesity lead to a risk of psychological and social problems related to a negative self-image, low self-esteem, increased depression disorder, inadequate sleep, and reduced adult life expectancy [13–15]. Furthermore, the economic impacts of childhood overweight and obesity increase medical cost, job absenteeism, annual drug usage, emergency room, outpatient cost of $14.1 billion, and inpatient cost of $236.6 million [16, 17].

Adolescents' overweight and obesity are rising alarmingly and approaching an epidemic in many developed countries and become a double burden for low- and middle-income courtiers [7, 18]. The global prevalence of obesity increased from 4% in 1975 to 18% in 2016, with an estimated 124 million being affected [3]. Reports affirmed that an estimated 43 million children are obese globally; of these, approximately 81% were from developing countries, half of which (18 million) were living in Asian countries [19].

In developing countries overweight and obesity increased from 8.1 to 13.4% in girls over thirty years [20]. In Asians, they increased from 9.8% to 11.7% [21], and about 8.7% to 31.4% developed overweight and obesity in Africa [22, 23]. A cross-sectional data from the Global School-Based Student Health Survey (GSHS) conducted in seven African countries showed that being overweight ranged from 8.7% (Ghana) to 31.4% (Egypt). Obesity rates ranged from 0.6% (Benin) to 9.3% (Egypt) [23]. In Ethiopia, the magnitude of overweight and obesity varies from place to place. For example, in a study done in Wolaita Sodo town 5.0% [24], Jimma town, Ethiopia 13.3% [25], and Addis Ababa, 8.2% [26] school adolescents were overweight and obese. This prevalence also varied in residences. The above literature showed that the prevalence of overweight and obesity increased from time to time.

Previous reports revealed that the socioeconomic status of the family, urbanization, sex, physical activity [27], dietary habits, meal frequency, breakfast skipping, and physical activities are considered important risk factors [28, 29]. Additional factors, which have a relative contribution are genetic variation, epigenetic and intrauterine exposures, transportation, could also be contributing factors [30, 31]. Despite efforts from the government of Ethiopia to reduce the prevalence of overweight and obesity [32], their prevalence in children and youth remains high [25]. In addition, there was a great variation and inconsistency in the prevalence and factors among the studies [33]. Therefore, this study aimed to assess the prevalence and associated factors of obesity and overweight among high school adolescents in Bahir Dar city.

## 2. Methods

### 2.1. Study Setting and Period

An institutional-based cross-sectional study was conducted from October 30, 2018, to November 30, 2018, among high school adolescents in Bahir Dar city. Bahir Dar is the capital city of the Amhara National Regional State located 563 km away from Addis Ababa, the capital city of Ethiopia. The city has one special zone and five subcities and private and eleven public secondary schools. In Bahir Dar city, there are 16,782 high school students: 14,483 are public and 2293 are private students [34].

### 2.2. Source and Study Population

All adolescent students aged 10–19 in Bahir Dar city enrolled from grades 9 to 12 for 2018/2019 academic years were the source populations. Students enrolling in the selected schools during the data collection period were considered the study population.

### 2.3. Inclusion and Exclusion Criteria

All students from grades 9 to 12 with the age ranges of 10–19 years attending during the data collection period were included in the study and severely ill students during the data collection period were excluded from the study.

### 2.4. Sample Size Determination

The sample size was calculated using a single proportion formula by considering the following assumptions: previous prevalence of overweight and obesity among high school students 16.7% [35], 95% confidence level, 4 of marginal error, and 1.5 of the design effect. Finally, a sample size of 551 was obtained by considering a 10% nonresponse rate.

### 2.5. Sampling Technique

Among the 18 secondary schools (16,782 total number of students) in Bahir Dar city, five were selected using the simple random sampling technique to achieve the primary sampling units. The total sample size was allocated to the five schools (8,263 students) proportionally, based on the number of students in each school. Of these, 551 students were selected by using a simple random sampling technique.

### 2.6. Data Collection Tool and Procedure

Sociodemographic data were collected using a structured questionnaire developed from similar literature [35, 36]. The questionnaire was initially prepared in English, translated into Amharic, the local language, and retranslated to English to maintain consistency. One-day training was given for 5 data collectors and 2 supervisors to create awareness of data collection, procedures, and the technique of how to collect data. The structured questionnaire was used to collect data on sociodemographic characteristics, learning hours, school feeding habits, use of soft drinks, feeding frequency in a day, a history of family obesity and chronic disease, transportation type, and physical exercise. A pretest was done among 5% of the total sample size similar to the study setting to ensure questioner consistency and quality. Daily data follow-up and checkup were done through the principal investigator and supervisors. In Bahir Dar city, 18 secondary schools were found; five were selected using the simple random sampling technique to achieve the primary sampling units. The calculated samples of students were recruited from selected schools based on the proportional number of their students.

### 2.7. Variables Measurement

Height was measured to the nearest 0.1 cm using a height measuring stadiometer in a standing position with a sliding headpiece. The subject stood up on the basal part of the device with feet together and shoulder, buttock, calf, and heels touching the vertical stand of the stadiometer. Or the study participant stands with their eyes in the Frankfurt horizontal plane. Weight was measured to the nearest 0.1 kg using beam balance. Body mass index for age (BAZ) was calculated using WHO AnthroPlus software and BMI for the age of Z scores +2 to +3 was classified as overweight, whereas BMI for age ≥3 Z score was considered as obese [30, 37].

School feeding habit is the provision of meals to students in class or take-home ration to families with children who attend school regularly [38].

The household wealth index was determined using the Principal Component Analysis (PCA) by considering household assets. First, variables coded between 0 and 1 were entered and analyzed using PCA; then, variables with commonality values of greater than 0.5 were used to produce factor scores. Finally, the factor scores were summed and ranked as poor, medium, and rich.

Anthropometric data were collected by recording the age, weight, and height of the participants according to WHO guidelines. The instruments were checked for accuracy before data collection started. The participants were measured with an adjusted weight and height scale by trained data collectors. Weight and height were adjusted to the nearest 0.1 g and 0.1 cm, respectively. BMI for age was calculated using AnthroPlus software and adolescents having BAZ above +1 standard deviation (SD) were considered as overweight and obese [30, 37].

### 2.8. Data Processing and Analysis

Clean coded data were entered into Epi Info version 3.5.3 software and exported to SPSS version 22 for further analysis. BMZ score was computed by using WHO AnthroPlus software. Data were analyzed using the bivariable and multivariable logistic regression model. The PCA was used to compute wealth index individuals. Descriptive statistical analysis was described by sentences, graphs, tables, frequencies, percentages, and mean and standard deviation. A bivariable and multivariable logistic regression analysis was performed to determine the association between the dependent variable and the independent variables.

Factors associated with overweight on bivariable were identified, and the variables with a *p* value of 0.2 and less were entered into the multivariable analysis for controlling potential confounders. The odds ratio was used to measure the strength of association and reported with 95% confidence intervals; *p* value <0.05 was considered to be statistically significant for overweight and obesity.

## 3. Results

### 3.1. Sociodemographic Characteristics

A total of 522 participants participated with a response rate of 94.74%. Most participants 82.6% (431) were public school students and more than half, 275 (52.7%), were males. The mean age of the respondents was 17 ± 1.4 years. Regarding religions, 425 (81.4%) were Orthodox Christian and 483 (92.5%) were living in urban residence. More than two-thirds of study participants could not use any device for transportation and one-third, 161 (31.6%), of adolescents were from the lowest wealth status family (Table 1).

### 3.2. Dietary, Lifestyle, and Parent History-Related Characteristics

Regarding the dietary habits of the study participants, more than one-fourth, 146 (28.0%), of participants were eating three times or less per day; more than, two-thirds, 360 (69.0%), of adolescents did not have school feeding habits; and the majority 455 (87.2%) of the participants took beverages. Only, 54 (10.3%) of the respondents had a family history of chronic diseases and 51 (9.8%) had a family history of obesity. Nearly, half of the respondents, 257 (49.2%), were engaged in physical exercise (Table 2).

### 3.3. Prevalence and Associated Factors of Overweight and Obesity

The overall prevalence of overweight and obesity was found to be 12.5% (95% CI: 9.6, 15.2%). Of this, 7.7% and 10.7% were overweight and obese, respectively. Adolescents, who were living in rich families (15.6%), developed overweight and obesity, and the most prevalence (17.6%) of overweight and obesity was observed within the age range of 10–14 years old.

Variables having *p* values of 0.20 or less in the bivariate logistic regression were fitted in the multivariate logistic regression to get the adjusted effect of each covariate. After adjusting for independent variables, maternal occupation and student's school feeding habits were significantly associated with overweight and obesity at a 95% confidence level and a *p* value of <0.05.

High school adolescents with self-employed mothers were about 4.6 times more likely to be overweight and obese (AOR: 4.57; 95% CI: 1.06, 19.78) compared to those having housewife mothers. In addition, adolescents having government-employed mothers were also almost 6.5 times more likely to be overweight and obese (AOR: 6.49; 95% CI: 1.96, 21.54). Those adolescents having school feeding habits reduced overweight and obesity by 54% (AOR: 0.44; 95% CI: 0.26, 0.76) (Table 3).

## 4. Discussion

Nutrition in the early stage has a role in social skills, physical appearance, emotional stability, and quality of life of adolescents. The economic growth, in terms of both lost productivity and increased burden of disease, is affected by malnutrition [39]. This study aimed to assess the prevalence and associated factors of overweight/obesity among school adolescents. The current study shows males were more likely than females to be overweight or obese (67% vs. 52%). The possible reason might be cultural practice can be the possible cause of overweight and obesity among male adolescents because males are free to purchase more junk food from mobile carts of street vendors' junky foods. In the contrast, female adolescents are more selective for food choice maintaining star-like body image as compared with males adolescents.

The overall prevalence of overweight and obesity among adolescents was 12.5%. This finding is supported by the studies in Jimma town, 13.3% [25], and Bahir Dar city, 11.9% [30]. This could be because both the previous studies and our study are done in a similar study setting; socioeconomic, cultural, dietary habit; and lifestyle-related characteristics of the study participants.

The current finding was lower than the research findings in Ghana, 16.4% [40], and China, 20.0% [41]. The reason for the variability may be attributed to different factors including the sociodemographic and economic differences (36). However, our finding is higher than the studies done in Nigeria, 7% [42], and Gondar town, 6.0% [43]. The disparity might be due to sociodemographic and economic variations and might be related to urbanization; thus, adolescents living in urban areas might have more overweight and obesity rates due to transportation modalities and cultural differences in dietary intake.

High school adolescents having self-employed and government-employed mothers were more likely to be overweight and obese compared to adolescents having housewife mothers. This finding is in line with a study done in the United States [44]. The possible reason might be that self-employed and government-employed mothers had probably better economic and educational status and a strong association with overweight and obesity. For the participants having better economic and educational status, getting access to affordable healthy foods might be increased. However, another study showed that economic status and overweight/obesity were inversely associated among adolescents and this magnitude increased with age and differed with gender [45]. Such an inverse association has been speculated due to behavioral factors associated with socioeconomic status. In addition to this, government and self-employed participants might be more educated and when parents are more educated, the awareness of their children's weight status is increased and they practice a healthier lifestyle. Alternatively, the educational level may represent a proxy for family wealth, making it easier for those families to take healthier turns [46].

Adolescents having school feeding habits reduced overweight and obesity. This finding is similar to a study done in French primary-school children [47]. This might be due to the fact that the school feeding program has reducing short-term hunger and enables children to receive a diversified diet based on the school feeding schedule. In addition, it has the role of preventing adolescents' purchasing unhealthy diet meal away from the home. Moreover, based on the meal of the school feeding habits, the energy intake and expenditure might be proportional to prevent over- and undernutrition in adolescents. Furthermore, the school environment has its own role in reducing unhealthy weight gain; it may be due to the fact that school has sport/physical exercise program of how to maintain health through playing football, tennis, basketball, and other types of exercise to reduce extra storage of energy in the body causing overweight and obesity. School might have media to deliver simple and clear nutrition information that will change unhealthy eating behaviors which can contribute to better nutritional outcomes.

This association between the risk of becoming overweight and obesity and eating at school has not previously been reported, although several studies confirm the relevance of the school as a means of influencing children's eating habits. However, a study done in South African showed that dietary habits and eating practices within the school were not found to be significantly associated with the likelihood of being overweight and obese among early and mid-adolescents [48].

### 4.1. Limitations of the Study

The limitation of this study is that it could not consider genetic influences, parental body mass index, and the health condition of participants. Another limitation might be recall bias that influences results as the participants could forget what they consumed and their level of physical activity. Finally, as dietary intake and physical activity were self-reported data, over- or underreporting may be a significant concern for this study.

## 5. Conclusion

The current study showed that the prevalence of overweight and obesity among school adolescents is a high public health problem. Overweight/obesity is significantly associated with maternal occupation school feeding habit. Therefore, more emphasis should be given to adolescents having mothers who are working on self- and government-employed students and students having school feeding habits.

## Figures and Tables

**Table 1:** Sociodemographic characteristics of school adolescents and their parents at Bahir Dar city high school (*n* = 522).

Characteristics	Frequency	Percentage
*Sex*		
Male	270	51.7
Female	252	48.3

*Age*		
Early	4	0.8
Middle	312	59.8
Late	206	39.5

*Residence of family*		
Urban	483	92.5
Rural	39	7.5

*Religion*		
Orthodox	425	81.4
Muslim	89	17.0
Protestant	8	1.5

*Grade level*		
9–10	262	50.2
11–12	260	49.8

*Father educational*		
Unable to read and write	75	14.4
Able to read and write	7	1.3
Primary	152	29.1
Secondary	93	17.8
Higher education	195	37.4

*Maternal educational*		
Unable to read and write	94	18.0
Able to read and write	10	1.9
Primary	147	28.2
Secondary	122	23.4
Higher education	149	28.5

*Father occupation*		
Farmer	68	13.0
Daily labour	37	7.1
Merchant	168	32.2
Self-employee	66	12.6
Government employee	183	35.1

*Maternal occupation*		
Farmer	266	51.0
Daily labour	25	4.8
Merchant	91	17.4
Self-employee	43	8.2
Government employee	97	18.6

*Wealth status*		
Poor	165	31.6
Medium	177	33.9
Rich	180	34.5

**Table 2:** Dietary, lifestyle, and parent history-related characteristics of high school adolescents at Bahir Dar city, northwest Ethiopia (*n* = 522).

Characteristics	Frequency	Percentage
*Feeding frequency*		
≤3 times	146	28.0
>3 times	376	72.0

*School feeding habit*		
Yes	162	31.0
No	360	69.0

*Beverage drinking*		
Yes	455	87.2
No	67	12.8

*Physical exercise*		
Yes	257	49.2
No	265	50.8

*Transportation type*		
Bicycle	71	13.6
Motor cycle	15	2.9
Automobile	91	17.4
Foot	345	66.1

*Family chronic disease history*		
Yes	54	10.3
No	468	89.7

*History of family obesity*		
Yes	51	9.8
No	471	90.2

**Table 3:** Factors associated with overweight and obesity among high school adolescents at Bahir Dar city, northwest Ethiopia.

Characteristics	Overweight/obesity		Crude odds ratio (95% CI)	Adjusted odds ratio (95% CI)
	Yes	No		
*Age in years*				
10–14	4 (23.5%)	13 (76.5%)	0.45 (0.14, 1.41)	0.37 (0.11, 1.29)
15–19	61 (12.1%)	444 (87.9%)	1	1

*Religion*				
Orthodox	46 (10.8%)	379 (89.2%)	1	1
Muslim	17 (19.1%)	72 (80.9%)	0.51 (0.28, 0.95)	0.58 (0.31, 1.10)
Protestant	2 (25.%)	6 (75.0%)	0.36 (0.07, 1.86)	0.33 (0.06, 1.79)

*Transportation type*				
Bicycle	4 (5.6%)	67 (94.4%)	2.26 (0.78, 6.52)	1.88 (0.64, 5.52)
Motor cycle	5 (33.3%)	10 (66.7%)	0.27 (0.09, 0.83)	0.45 (0.13, 1.51)
Automobile	15 (16.5%)	76 (83.5%)	0.68 (0.36, 1.29)	0.84 (0.41, 1.72)
Foot	41 (11.9%)	304 (88.1%)	1	1

*School type*				
Government	47 (10.9%)	384 (89.1%)	1	1
Private	18 (19.8%)	73 (80.2%)	0.49 (0.27, 0.90)	0.78 (0.33, 1.81)

*Learning hour*				
≤7 hours/day	53 (11.4%)	411 (88.6%)	1	1
>7 hours/day	12 (20.7%)	46 (79.3%)	0.49 (0.25, 0.99)	0.68 (0.29, 1.59)

*Father occupation*				
Farmer	10 (14.7%)	58 (85.3%)	1	1
Daily labour	9 (24.3%)	28 (75.7%)	0.54 (0.19, 1.47)	0.55 (0.17, 1.78)
Merchant	19 (11.3%)	149 (88.7%)	1.35 (0.59, 3.08)	1.99 (0.76, 5.22)
Self-employee	11 (16.7%)	55 (80.3%)	0.86 (0.34, 2.19)	1.12 (0.40, 3.13)
Government employee	16 (8.7%)	167 (91.3%)	1.80 (0.77, 4.19)	1.16 (0.43, 2.91)

*Mother occupation*				
Housewife	44 (16.5%)	222 (83.5%)	1	1
Daily labour	3 (12.0%)	22 (88.0%)	1.45 (0.42, 5.07)	1.51 (0.43, 5.33)
Merchant	13 (14.3%)	78 (85.7%)	1.19 (0.61, 2.33)	1.25 (0.64, 2.47)
Self-employee	2 (4.7%)	41 (95.3%)	4.06 (0.95, 17.42)	4.57 (1.06, 19.78)*∗*
Government employee	3 (3.1%)	94 (96.9%)	6.21 (1.88, 20.49)	6.49 (1.96, 21.54)*∗*

*Wealth status*				
Poor	21 (12.7%)	144 (87.3%)	1	1
Medium	15 (8.5%)	162 (91.5%)	1.57 (0.78, 3.17)	1.5 (0.72, 3.14)
Rich	29 (16.1%)	151 (83.9%)	0.76 (0.41, 1.39)	0.89 (0.45, 1.81)

*School feeding habit*				
Yes	30 (18.5%)	132 (81.5%)	0.47 (0.28, 0.80)	0.44 (0.26, 0.76)*∗*
No	35 (9.7%)	325 (90.3%)	1	1

*∗*Significant at *p* value of ≤0.05.

## Data Availability

All the data are available from the corresponding author upon request.

## Abbreviations

AOR: Adjusted odds ratio
BDAC: Bahir Dar administration city
BMI: Body mass index
BDRAC: Bahir Dar academy
CDC: Center of disease control
CI: Confidence interval
CVD: Cardiovascular disease
DALY: Disability adjusted life year
DC: Data collector
DDS: Dietary diversity score
DM: Diabetes mellitus
ECC: Ethical clearance committee
EDHS: Ethiopian demographic health survey
FMOH: Federal ministry of health
KG: Kilogram
MAM: Moderate acute malnutrition
MUAC: Midupper arm circumference
SAM: Severe acute malnutrition
WHO: World Health Organization.
